# A deep ensemble learning-based automated detection of COVID-19 using lung CT images and Vision Transformer and ConvNeXt

**DOI:** 10.3389/fmicb.2022.1024104

**Published:** 2022-11-04

**Authors:** Geng Tian, Ziwei Wang, Chang Wang, Jianhua Chen, Guangyi Liu, He Xu, Yuankang Lu, Zhuoran Han, Yubo Zhao, Zejun Li, Xueming Luo, Lihong Peng

**Affiliations:** ^1^School of Computer Science, Hunan University of Technology, Zhuzhou, China; ^2^Geneis (Beijing) Co., Ltd., Beijing, China; ^3^Hunan Storm Information Technology Co., Ltd., Changsha, China; ^4^High School Attached to Northeast Normal University, Changchun, China; ^5^No. 2 Middle School of Shijiazhuang, Shijiazhuang, China; ^6^School of Computer Science, Hunan Institute of Technology, Hengyang, China; ^7^College of Life Sciences and Chemistry, Hunan University of Technology, Zhuzhou, China

**Keywords:** COVID-19, CT scan image, deep ensemble, Vision Transformer, ConvNeXt

## Abstract

Since the outbreak of COVID-19, hundreds of millions of people have been infected, causing millions of deaths, and resulting in a heavy impact on the daily life of countless people. Accurately identifying patients and taking timely isolation measures are necessary ways to stop the spread of COVID-19. Besides the nucleic acid test, lung CT image detection is also a path to quickly identify COVID-19 patients. In this context, deep learning technology can help radiologists identify COVID-19 patients from CT images rapidly. In this paper, we propose a deep learning ensemble framework called VitCNX which combines Vision Transformer and ConvNeXt for COVID-19 CT image identification. We compared our proposed model VitCNX with EfficientNetV2, DenseNet, ResNet-50, and Swin-Transformer which are state-of-the-art deep learning models in the field of image classification, and two individual models which we used for the ensemble (Vision Transformer and ConvNeXt) in binary and three-classification experiments. In the binary classification experiment, VitCNX achieves the best recall of 0.9907, accuracy of 0.9821, F1-score of 0.9855, AUC of 0.9985, and AUPR of 0.9991, which outperforms the other six models. Equally, in the three-classification experiment, VitCNX computes the best precision of 0.9668, an accuracy of 0.9696, and an F1-score of 0.9631, further demonstrating its excellent image classification capability. We hope our proposed VitCNX model could contribute to the recognition of COVID-19 patients.

## Introduction

In March 2020, the World Health Organization declared COVID-19 as an international pandemic disease due to its rapid and strong transmission (Cascella et al., 2022). Until 22 April 2022, the pandemic has caused about 6.213 million deaths worldwide, over 505.8 million people have been infected with this virus, and there are up to ~700 thousand new cases within 24 h of that time (Geneva: World Health Organization, 2020; Wang et al., 2021). Different from SARS, the new coronavirus did not disappear quickly or cause limited losses (Stadler et al., 2003). On the contrary, its Delta and Omicron variants induced new pandemics worldwide after multiple mutations (Vasireddy et al., 2021; V'kovski et al., 2021; Yu et al., 2021; Del Rio et al., 2022). It has also caused a sustained impact on the global economy. Long-term shutdowns left many people unemployed. Many countries enforced lockdowns during periodical outbreaks, which resulted in a global economic recession (Alshater et al., 2021; Padhan and Prabheesh, 2021). Although vaccines have been researched and developed to prevent COVID-19 transmission to a certain extent, there is still a need to adopt various methods to detect the virus and prevent its spread.

As a highly contagious respiratory disease, the clinical symptoms of COVID-19 are similar to the common flu and common pneumonia, for instance, coughing, dyspnea, dizziness, and some mild symptoms (Zhang et al., 2020). But the patient infected by the novel coronavirus may deteriorate into fatal acute respiratory distress syndrome in a very short period of time (Guan, 2020). As a result, it greatly increases the difficulty of its early detection and places higher demands on the healthcare system for its treatment. Therefore, the efficient and accurate identification of COVID-19 in patients has become a key to preventing its spread. The nucleic acid test is currently the most widely used due to its high accuracy, simple operation, and low cost (Tahamtan and Ardebili, 2020). But the paucity of standard laboratory environments with specially trained staff has limited the entire testing process.

As an alternative, the non-invasive detection technology, Computed Tomography (CT) provides a new rapid detection method for detecting COVID-19. After the patient has undergone a lung CT scan, experienced radiologists can quickly find typical lesions in the patient's lungs, such as ground-glass opacity, consolidation, and interlobular interstitial thickening by reading the CT images (Chung et al., 2020; Xu et al., 2020). We can also detect COVID-19 in a short time by combining patients' clinical symptoms and investigating recent social situations using epidemiological survey methods. It can help medical workers and epidemic management departments to quickly deal with patients and deploy new prevention and control strategies, and thus intervene in the treatment of patients as early as possible to control its contagion.

However, during the initial stage of the epidemic outbreak, the massive influx of patients often means medical staff and healthcare professionals have to work 24 h a day, which has a bad effect on the physical and mental health of doctors and affects the accuracy and efficiency of the medical diagnosis (Zhan et al., 2021). Alternatively, artificial intelligence technology is a quite efficient strategy and obtains wide application in various fields (Chen et al., 2019; Liu et al., 2021a, 2022a,b; Tang et al., 2021; Wang et al., 2021; Zhang et al., 2021; Liang et al., 2022; Sun et al., 2022; Yang et al., 2022), and can be used to complement the work of radiologists. It can efficiently assist medical staff in judging symptoms, for example, pre-classifying pathological images or predicting sampling results, and thus can greatly reduce their working intensity. Particularly, deep learning has achieved optimal performance in medical image processing (Munir et al., 2019). For instance, Sohail et al. (2021) used a modified deep residual neural network to detect pathological tissue images of breast cancer and implemented automated tumor grading by detecting cell mitosis. Similarly, Codella et al. (2017) introduced a deep ensemble model for pathological image segmentation of skin cancer and the detection of melanoma to improve the detection efficiency of skin cancer. Dou et al. (2016) established a three-dimensional multi-layer convolution model to detect pulmonary nodules in lung stereoscopic CT images, thereby reducing the false positive rate of automated pulmonary nodule detection. Farooq and Hafeez (2020) proposed a ResNet-based COVID-19 screening system to assist radiologists to diagnose. Aslan et al. (2021) developed a new type of COVID-19 infection detection system based on convolutional neural networks (CNN) by combining the long short-term memory (LSTM) network model. These methods effectively improved the identification performance of COVID-19-related CT images. In this paper, we propose a deep-learning ensemble model by integrating Vision Transformer (Dou et al., 2016) and ConvNeXt (Liu et al., 2022c) to effectively improve the prediction accuracy of COVID-19-related CT images.

## Materials and methods

### Materials

We constructed a comprehensive dataset by integrating and screening data from three lung CT datasets (Soares et al., 2020; Yang et al., 2020). Dataset 1 contained a total of 4,171 images, where 2,167 images were from COVID-19 patients, 757 were from healthy people, and 1,247 were from other pneumonia patients. Dataset 2 contained a total of 2,481 images, where 1,252 images were from COVID-19 patients, and 1,229 were from healthy people; both datasets 1 and 2 were from São Paulo, Brazil. Dataset 3 was from Wuhan, China, and included 746 CT images, of which 349 were from COVID-19 patients and 397 were from healthy people. Using these datasets we constructed an integrated dataset with a total of 7,398 CT images, which had 3,768 CT images of COVID-19 patients, 2,383 healthy CT images, and 1,247 CT images of other pneumonia patients.

### Methods

We investigated various CNN and transformer models and chose Vision Transformer and ConvNeXt as the basic classifier of the ensemble model.

#### Vision transformer

Transformers have been widely used in the natural language processing field since it was proposed in 2017 (Vaswani et al., 2017). It constructs basic decoder units by connecting the feed-forward neural network and the self-attention mechanism (Bahdanau et al., 2014), as well as adding an encoder-decoder self-attention layer between the two network structures. It creates a brand-new structure that differs from CNN while obtaining relatively high accuracy. The self-attention mechanism used in the transformer first converts the input text into an embedding vector based on word embedding progress. Next, the obtained embedding vectors are used as inputs (named Queries, Keys, and Values) of the self-attention mechanism by a series of multiplication operations. Finally, the output of the self-attention layer is computed using Equation (1) and is fed to the next fully connected layer.


(1) $$\begin{array}{l} \text{Attention}\left(Q,K,V\right)=\text{softmax}\left(\frac{QK^{T}}{\sqrt{d_{k}}}\right)V\\ d_{k}=\dim\left(K\right) \end{array}$$

In 2020, Dosovitskiy et al. built Vision Transformer for image classification. It achieved powerful classification ability comparable to the top CNN models on multiple datasets (CIFAR-100, ImageNet, etc.) (Dosovitskiy et al., 2020).

**Figure 1:**
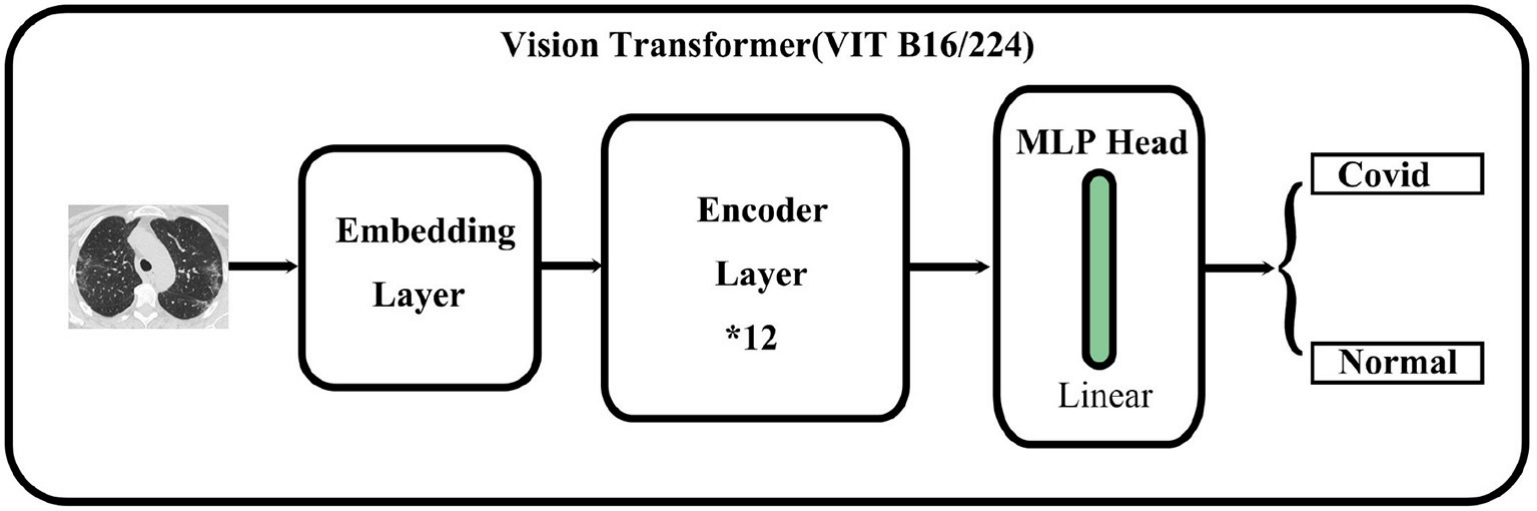
Concise structure of Vision Transformer.

As shown in Figure 1, the main architecture of the Vision Transformer model is mainly composed of three parts: First is the embedding layer which is used to convert an image into a vector that the transformer encoder can recognize. It also plays a role in embedding position information. The second is the transformer encoder layer which is used to extract features. Finally, a multi-layer perceptron head is used to feature dimension reduction and classify images.

#### The embedding layer

**Figure 2:**
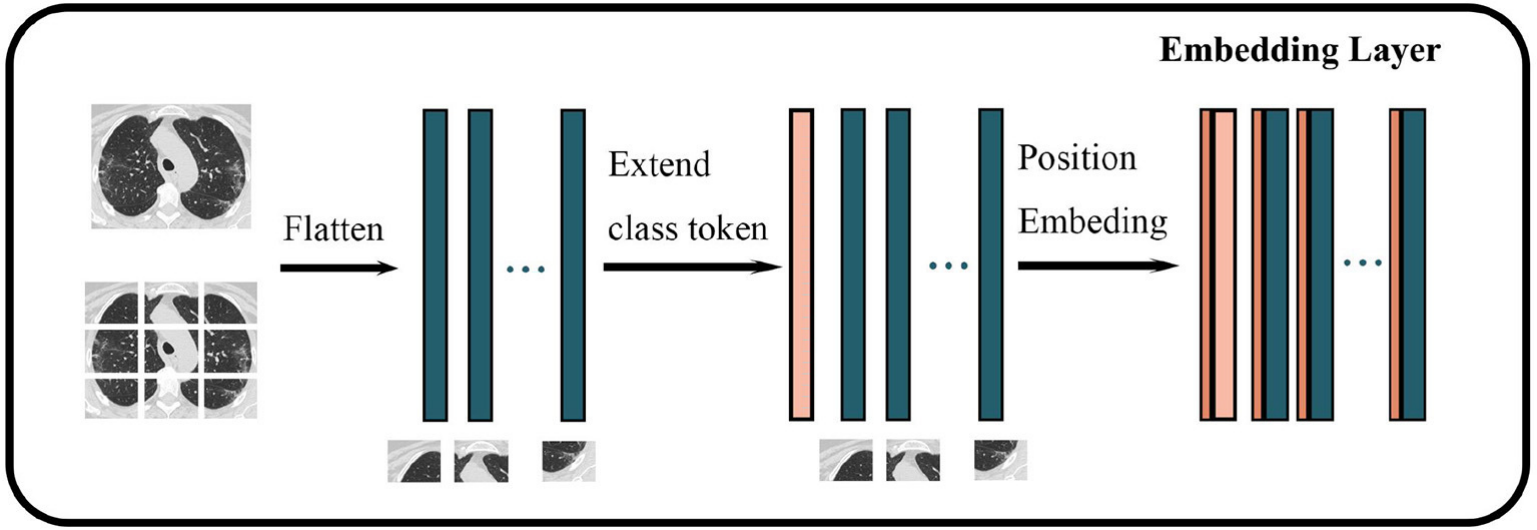
Structure of embedding layer in Vision Transformer. The darker green wider rectangles represent the flattened feature vector of each block of an image, while the pink wider rectangles represent the feature vectors corresponding to classes, and the brown narrower rectangle represents the spatiotemporal information of the image.

We used Vision Transformer-B/16-224 to classify COVID-19-related images. The procedure for embedding the layer is shown in Figure 2. First, an original image is resized to the following dimensions: 224^*^224^*^3. Second, the image is segmented into blocks of 16^*^16^*^3 according to the VIT-B/16-224 configuration, thereby generating 14^*^14 = 196 (224/16 = 14) blocks. Third, each block is mapped on a 768-dimensional vector through linear mapping. Finally, a matrix of 196^*^768 size is obtained as the basic input token.

In the original transformer model, all vectors need to embed position vectors to represent the spatiotemporal information of the original input. Similarly, Vision Transformer takes the location information as a trainable parameter and adds it to the token after the image is converted into a vector. The token is extended by one dimension, and a trainable parameter that represents the class or label is added to this new dimension to represent the original class or label of the token for training. The obtained final vector is input into the Transformer Encoder as a token.

#### Transformer encoder layer

**Figure 3:**
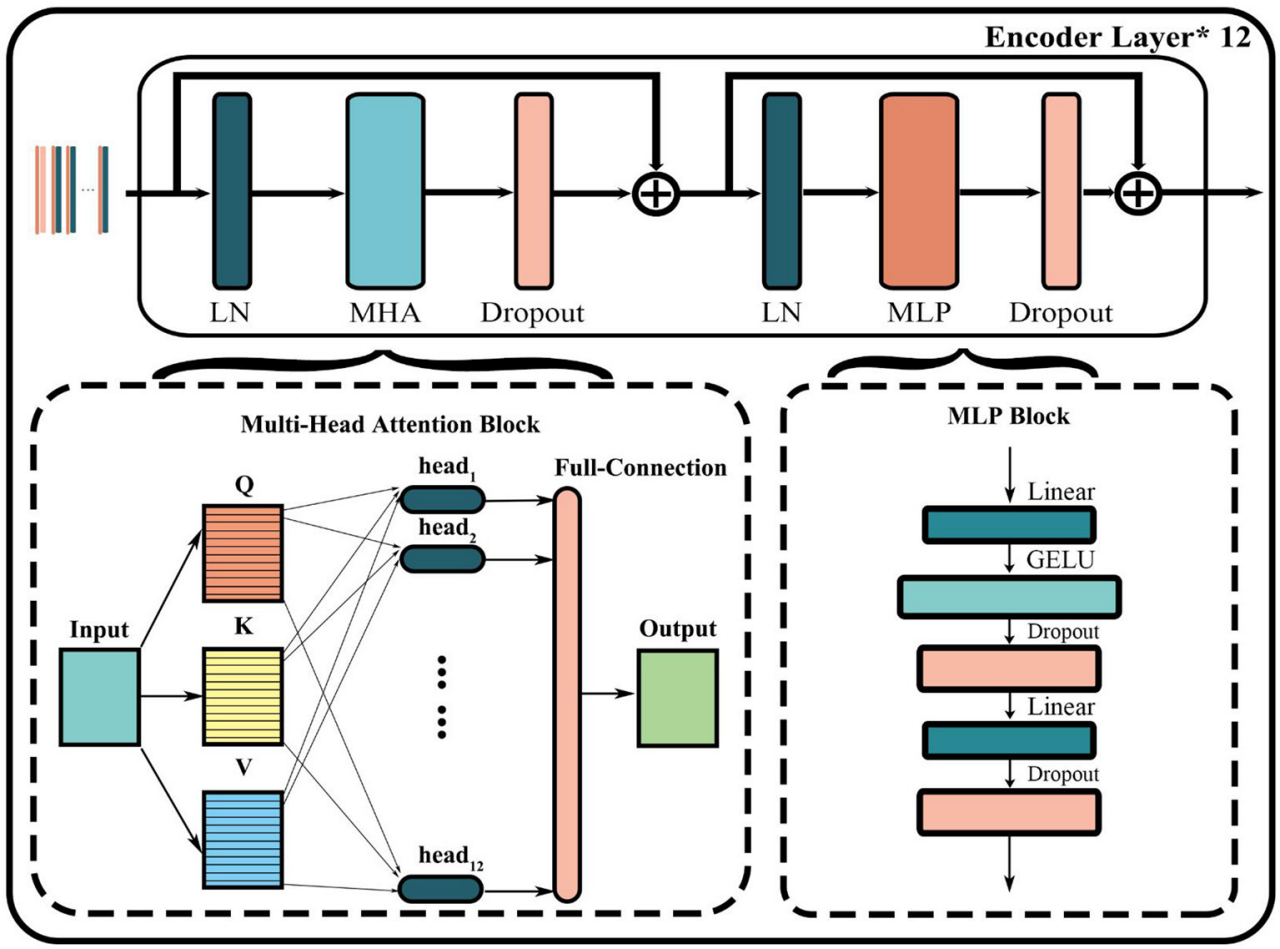
Structure of encoder layer in Vision Transformer.

As shown in Figure 3, the encoder layer mainly includes layer normalization (LN), multi-head attention (MHA) block, dropout, and multi-layer perceptron (MLP) block. The core of this structure is the parallel attention mechanism processing layer called multi-head attention. First, the input token matrix is normalized through layer normalization. Second, three matrices *Q*, *K*, and *V* are obtained by multiplying *W*^*Q*^and *W*^*K*^, *which* are the same as the self-attention module. Third, *Q*, *K*, and *V* are divided into a matrix equal to the number of heads h by multiples of $W_{i}^{Q},W_{i}^{K},W_{i}^{V}$. The corresponding *Q*_*i*_, *K*_*i*_, *V*_*i*_ matrix of each head is then used to compute the respective attention score using Equation (2):


(2) $$\begin{array}{l} {\text{head}}_{i}=\text{Attention}\left(QW_{i}^{Q},KW_{i}^{K},VW_{i}^{V}\right)\\ W_{i}^{Q}\in {\mathbb{R}}^{d_{model}\times d_{q}},W_{i}^{K}\in {\mathbb{R}}^{d_{model}\times d_{k}},W_{i}^{V}\in {\mathbb{R}}^{d_{model}\times d_{v}},\\ d_{q}=d_{k}=d_{v}=d_{model}/h \end{array}$$

Finally, the output of the MHA layer is obtained by concatenating all heads and multiplying a matrix-like full connection using Equation (3):


(3) $$\begin{array}{l} \text{MultiHead}\left(Q,K,V\right)=\text{Concat}\left({\text{head}}_{1},\ldots ,{\text{head}}_{h}\right)W^{o}\\ W^{o}\in {\mathbb{R}}^{hd_{v}\times d_{model}} \end{array}$$

The output of the entire transformer encoder layer can be obtained through a residual connection both before and after the MHA and MLP layers. And the encoder layer of the entire model is usually formed by stacking multiple transformer encoders.

#### MLP head

The main role of the MLP head is to obtain the high-dimensional features and obtain the final classification result. The outputs of the transformer encoder layer (197^*^768 in VIT B16/224) are used to compute the classification probability of an image. That is, the output of the transformer encoder layer is a 197^*^768 matrix, whose sizes are the same as the input of the transformer encoder layer. Finally, only one 768-dimensional vector is used as the input for the MLP head to obtain the classification result of an image corresponding to the matrix.

#### ConvNeXt

CNN is a classic neural network structure. Lenet was used for handwritten digit recognition as the earliest convolutional neural network model (LeCun et al., 1989). Due to the limitation of the lack of computer performance and the difficulty of collecting large-scale datasets in the 1990s, CNN did not achieve outstanding results in the 20 years that followed. In 2012, Krizhevsky et al. (2012) proposed the AlexNet CNN model, which defeated all image classification models at the ILSVRC2012 competition (Russakovsky et al., 2015). The following CNN models, for instance, VGGNet (Simonyan and Zisserman, 2014) and GoogleNet (Szegedy, 2015), have become prevalent in many AI application fields. The concept of residual and bottleneck layer proposed by the ResNet (He et al., 2016) model in 2015 again improved the performance of CNN. It effectively avoids the gradient problem caused by deeper layers. The generative adversarial network (GAN) proposed by Goodfellow et al. (2014) divided the network into two parts including generation and discriminator based on game theory to achieve better performance through iterative evolutions.

Since the transformer structure came into being in 2020, CNN has not become obsolete. On the contrary, the ConvNeXt network was introduced. ConvNeXt absorbs the advantages of multiple transformer structures in the network structure setting and parameter selection. It outperformed the most powerful transformer model named swin-transformer (Liu et al., 2021a,b) on the ImageNet-1K dataset by adjusting training parameter settings, optimizer, and convolution kernel sizes.

**Figure 4:**
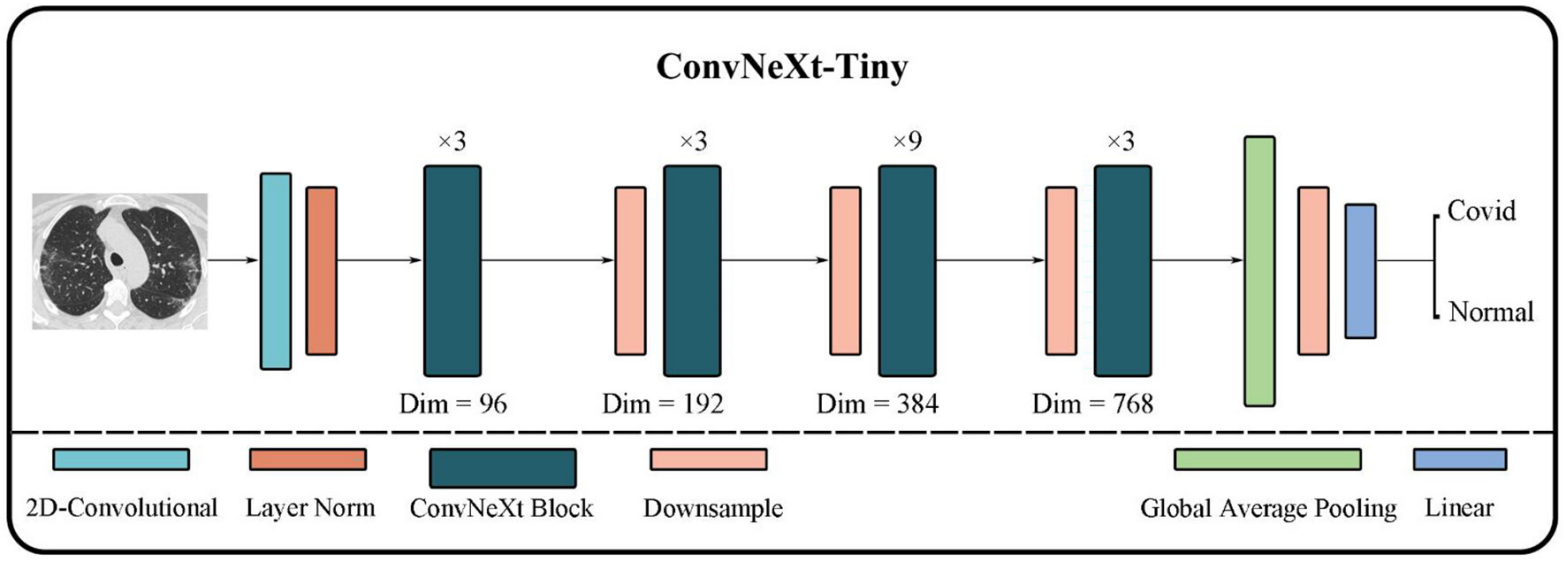
Concise structure of ConvNeXt.

As shown in Figure 4, ConvNeXt has a pretty concise structure. Its performance is greatly improved to the original ResNet although it is quite similar to ResNet. Moreover, it not only demonstrates better performance than many classic CNN models but also outperforms many transformer models.

First, ConvNeXt starts training ResNet-50 using techniques similar to training transformer models, such as better optimizers, more efficient hyper-parameter settings, and new data augmentation methods. Second, various new optimization strategies are gradually applied to optimize the model, for instance, setting new layer numbers and larger convolution kernels. And eventually, ConvNeXt outperforms the transformer model on the ImageNet-1K dataset.

**Figure 5:**
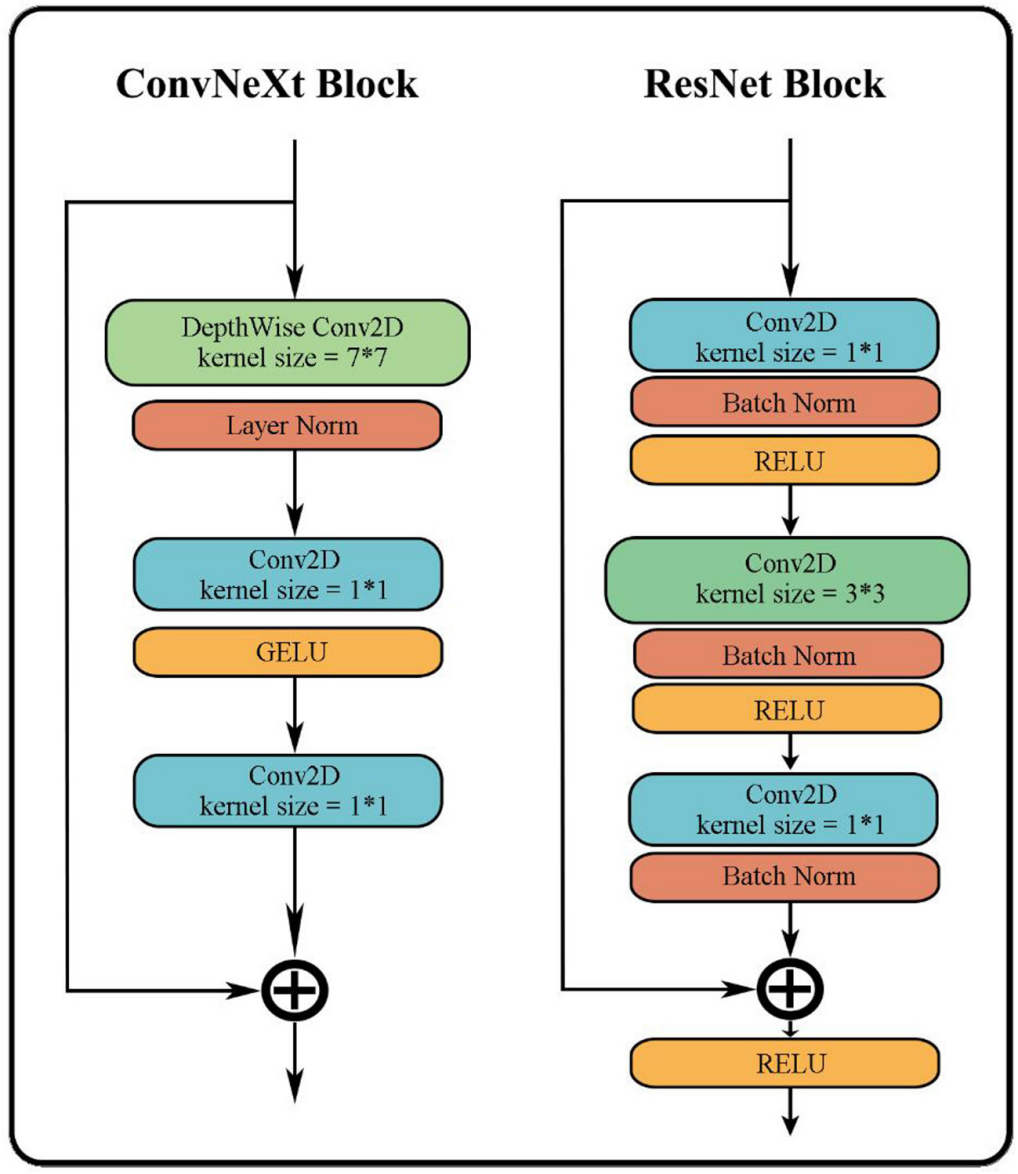
Differences between ConvNeXt and ResNet in bottleneck.

The overall structure of ConvNeXt is very similar to ResNet-50. It includes the feature extraction layer of the head, the middle layer where the bottleneck structure of four different dimensions is separately stacked, and the final high-dimensional feature classification layer. However, the strategy of stacking and the interior of each layer has undergone several changes. The changes include: (i) In each stage of the original ResNet-50, the stacking number of each block is 3:4:6:3; in ConvNeXt this has been revised to 3:3:9:3, which is similar to the block stacking of the transformer model. (ii) In the block of ResNet-50, the bottleneck design is to reduce the dimension first, then feature extraction, and finally increase the dimension. However, as shown in Figure 5, the bottleneck in ConvNeXt is designed to run feature extraction first, then reduce the dimension, and finally increase the dimension. (iii) It has modified the size of the convolution kernel to 7^*^7 from the ResNet 3^*^3. (iv) Its activation function has also been replaced from ReLU to GELU, and cut back the usage count of activation functions. (v) Its normalization has changed to layer normalization from batch normalization as well as reduced usage count of normalization. The performance of ConvNeXt has gradually improved and even outperforms the VIT through the above five strategies and a few other settings including new parameters, structures, and functions.

#### Ensemble

**Figure 6:**
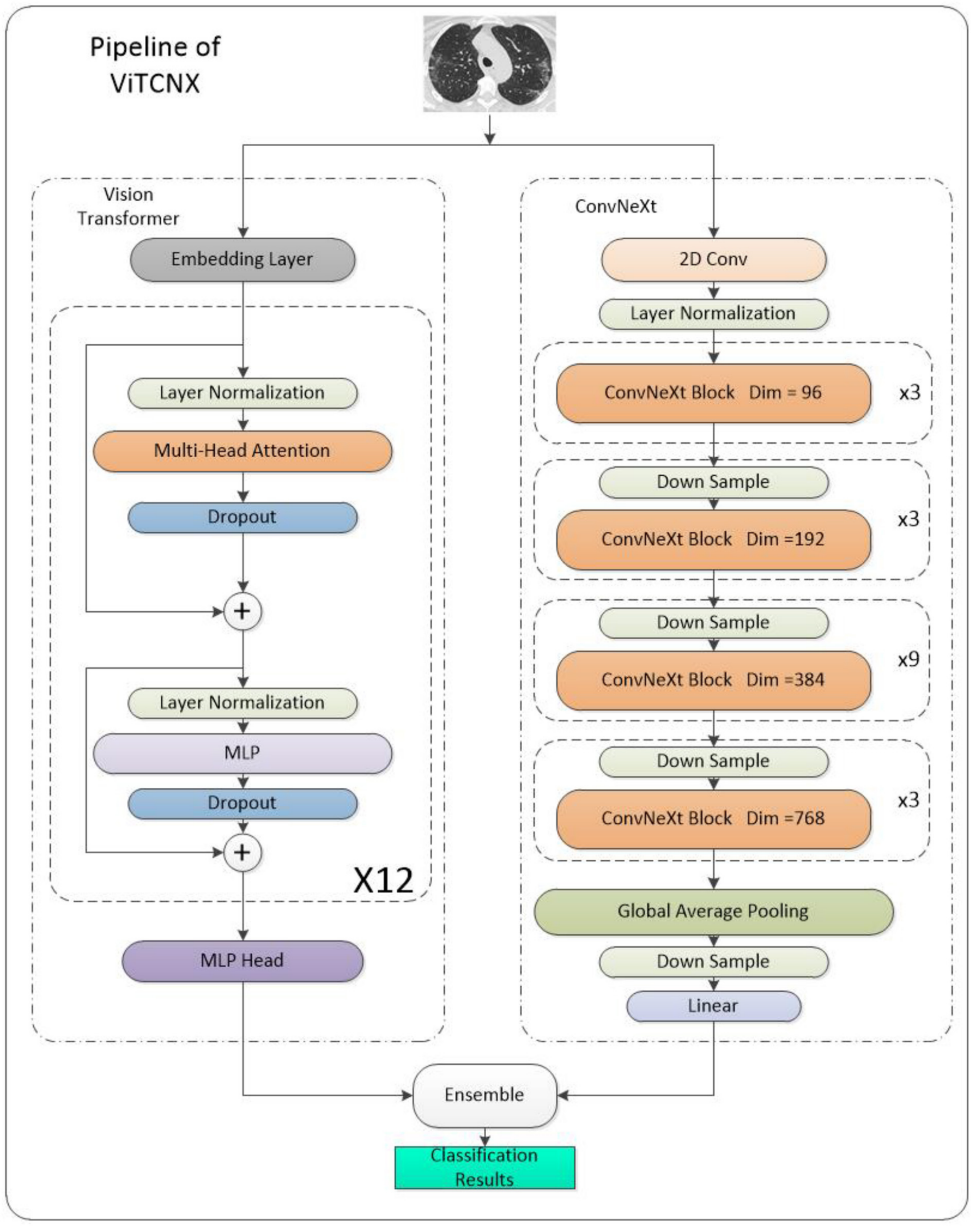
Pipeline of ViTCNX.

As shown in the pipeline in Figure 6, we can obtain the final classification results by integrating the results of the Vision Transformer and ConvNeXt based on the soft voting mechanism using Equation (4):


(4) $$\begin{array}{l} S_{f}=\alpha S_{v}+\left(1-\alpha \right)S_{c} \end{array}$$

Where *S*_*v*_ and *S*_*c*_ denote the classification scores from Vision Transformer and ConvNeXt for all images, respectively.

## Results

### Experimental evaluation and parameter settings

We used six metrics to evaluate the performance of all classification models, that is, precision, recall, accuracy, F1-score, AUC, and AUPR. These six evaluation metrics are defined as follows:


(5) $$\begin{array}{l} \text{Precision}=\;\frac{\text{TP}}{\text{TP}+\text{FP}} \end{array}$$

(6) $$\begin{array}{l} \text{Recall}=\;\frac{\text{TP}}{\text{TP}+\text{FN}} \end{array}$$

(7) $$\begin{array}{l} \text{Accuracy}=\;\frac{\text{TP}+\text{TN}}{\text{TP}+\text{TN}+\text{FP}+\text{FN}} \end{array}$$

(8) $$\begin{array}{l} \text{F}1-\text{Score}=\;\frac{2^{*}{\text{Precision}}^{*}\text{Recall}}{\text{Precision }+\text{ Recall}} \end{array}$$

(9) $$\begin{array}{l} \text{TPR}\left(\text{Ture Positive Rate}\right)=\;\frac{\text{TP}}{\text{TP}+\text{FN}} \end{array}$$

(10) $$\begin{array}{l} \text{FPR}\left(\text{False Positive Rate}\right)=\;\frac{\text{FP}}{\text{TN}+\text{FP}} \end{array}$$

AUC is the area under the TPR-FPR curve. AUPR is the area under the precision-recall curve. For COVID-19-related image binary classification, precision means the proportion of images that are COVID-19-related images in the dataset and are predicted to be COVID-19-related among all the predicted COVID-19 images. Recall represents the proportion of images that are COVID-19-related images in the dataset and are predicted to be COVID-19-related among all COVID-19-related images in the dataset. Accuracy represents the proportion that is correctly predicted. F1-Score, AUC, and AUPR are comprehensive metrics that consider precision, recall, and FPR.

To investigate the performance of our proposed ViTCNX model in different classification situations, we conducted experiments under binary classification and three-class classification, respectively. In the ViTCNX, the dataset was randomly initialized with seed = 8. ConvNeXt uses ConvNeXt_tiny to construct and initialize parameters, and its initial learning rate was set to 5e-4, and the initial weight adopted the convnext_tiny_1k_224_ema. The Vision Transformer uses vit_base_patch16 to construct and initialize parameters, and its initial learning rate was set to 1e-3. It adopted the initial weight vit_base_patch16_224_in21k. In all image classification algorithms, the training epoch and the batch size were set to 100 and 8, respectively. DenseNet, ResNet-50, Swin Transformer, and EfficinetNetV2 used densenet121, resnet50-pre, swin_tiny_patch4_window7_224, and pre_efficientnetv2-s to initialize their weight parameters, respectively. The corresponding learning rates were 1e-3, 1e-4, 1e-4, and 1e-3, respectively. ViTCNX used the same parameter settings as individual Vision Transformer and ConvNeXt. After comparing the image classification ability under different values of α, we set α = 0.6 where ViTCNX computed the best performance.

### Binary classification for CT images

**Figure 7:**
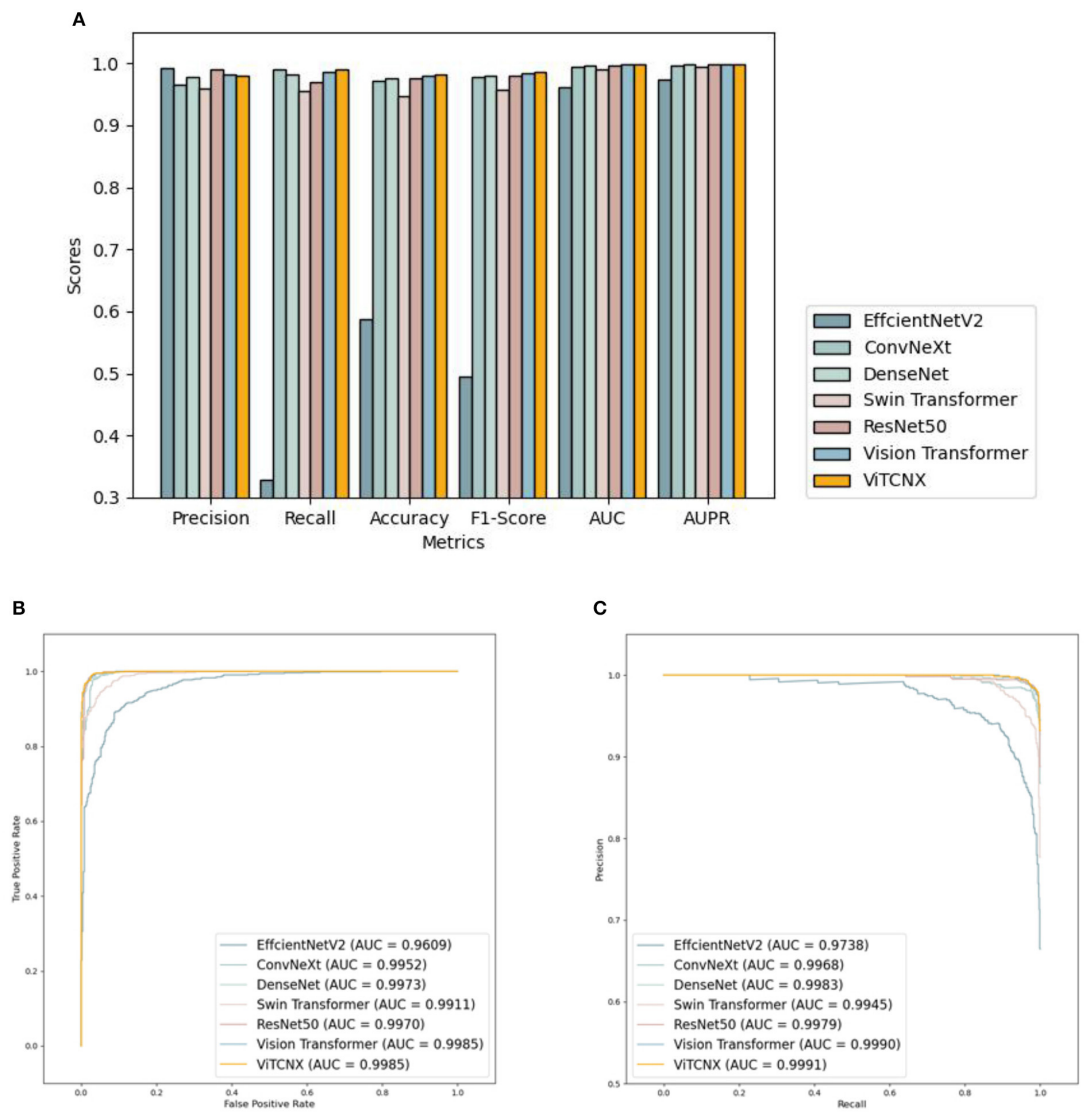
**(A)** The performance comparison of VitCNX and six other models for COVID-19 in binary classification problems; **(B,C)** The AUC and AUPR values of VitCNX and six other models for COVID-19 in binary classification problems.

Under the binary classification of images, there were a total of 6,151 CT images, including 3,768 CT images from COVID-19 patients and 2,383 CT images from healthy individuals. The 6,151 images were divided into a ratio of 0.8:0.2. Consequently, 4,922 images were used as the training set, including 3,015 COVID-19-related images and 1,907 CT images from healthy individuals. The remaining 1,229 images were used as the test set, including 753 COVID-19-related CT images and 476 healthy images. We compared our proposed ViTCNX model with four state-of-the-art image classification algorithms, that is, DenseNet (Huang et al., 2017), ResNet-50, Swin Transformer, and EfficinetNetV2 (Tan and Le, 2021). In addition, ViTCNX was also compared with the two individual models it was comprised of, that is, Vision Transformer and ConvNeXt. The results are shown in Table 1. The bold font in each column represents the best performance computed by the corresponding method among the above seven methods. Table 1 and Figure 7 show the precision, recall, accuracy, F1-score, AUC, and AUPR values and curves of these models.

**Table 1:** Performance of ViTCNX and the other six models under the binary classification.

**Metrics**	**Precision**	**Recall**	**Accuracy**	**F1-score**	**AUC**	**AUPR**
EfficientNetV2	**0.9920**	0.3293	0.5875	0.4945	0.9609	0.9738
ConvNeXt	0.9650	0.9894	0.9715	0.9770	0.9952	0.9968
DenseNet	0.9788	0.9814	0.9756	0.9801	0.9973	0.9983
Swin Transformer	0.9587	0.9548	0.9471	0.9568	0.9911	0.9945
ResNet-50	0.9892	0.9695	0.9748	0.9792	0.9970	0.9979
Vision Transformer	0.9815	0.9854	0.9797	0.9834	0.9985	0.9990
ViTCNX	0.9803	**0.9907**	**0.9821**	**0.9855**	**0.9985**	**0.9991**

Bold values means the highest score under this metric.

From Table 1 and Figure 7, we can find that ViTCNX obtained the best recall, accuracy, F1-score, AUC, and AUPR, significantly outperforming the other six methods. EfficientNetV2 achieved the best score of precision. This result is consistent with the prediction results on the confusion matrix. In the experiments, EfficientNetV2 computed higher precision than ViTCNX. The reasons may be that different models perform very differently on different parameter settings, different datasets, and different sizes, which have a significant impact on the classification performance of the model. In particular, ViTCNX outperforms its two individual models, Vision Transformer and ConvNeXt, demonstrating that an ensemble of single classification models can improve image identification performance. Figures 7B,C show the AUC and AUPR values obtained by the seven models. ViTCNX outperforms the other six models, elucidating that it can effectively classify related CT images as COVID-19-related or not.

### Three-classification for CT images

**Figure 8:**
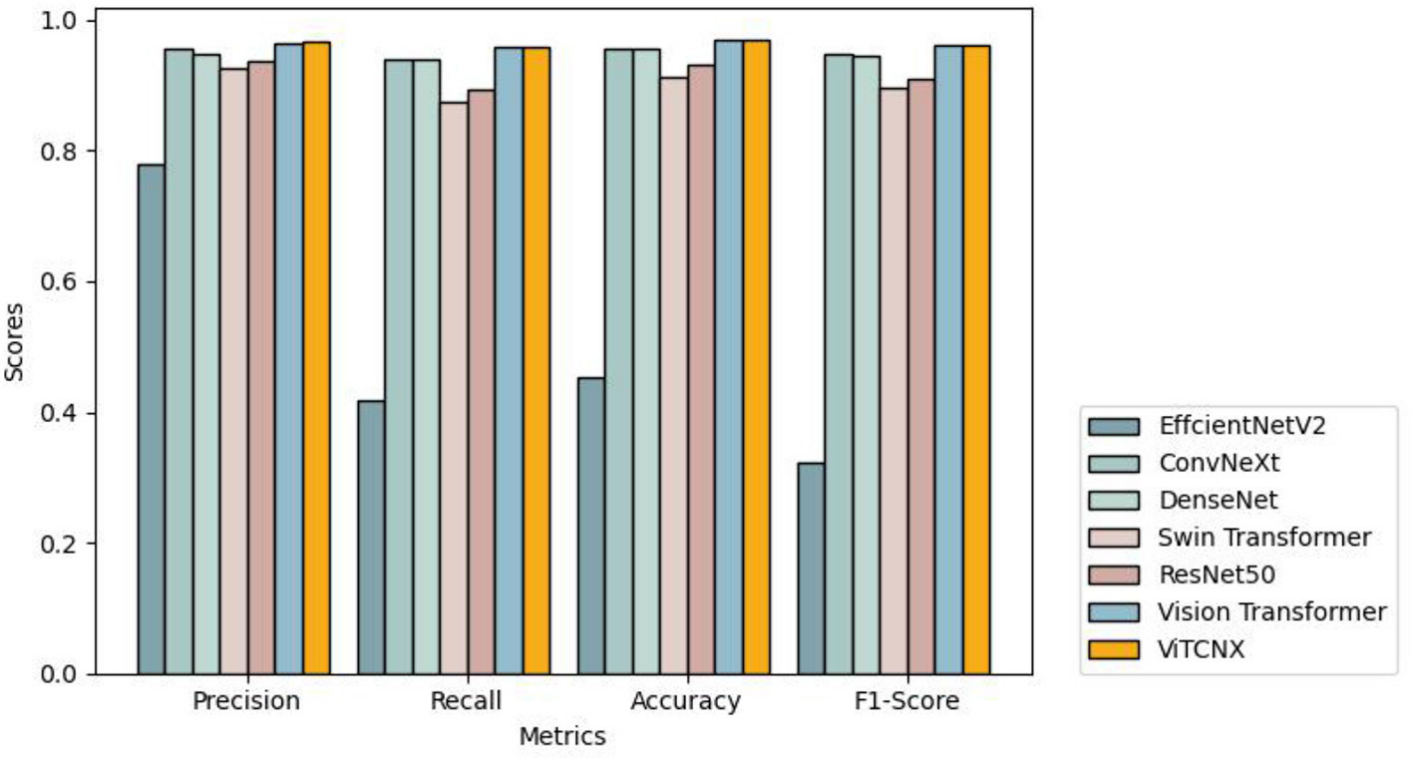
The performance of VitCNX and six other models for three-classification problem.

To further investigate the performance of the seven models under the three-classification challenge, we considered a total of 7,398 CT images, including 3,768 images from COVID-19 patients, 2,383 from healthy individuals, and 1,247 from other pneumonia patients. The 7,398 images were divided in a ratio of 0.8:0.2, resulting in 5,920 images in the training set and 1,478 images in the test set. The 5,920 images in the training set consisted of 3,015, 1,907, and 998 images from COVID-19 patients, healthy individuals, and other pneumonia patients, respectively. The 1,478 images in the test set consisted of 753, 476, and 249 images from COVID-19 patients, healthy individuals, and other pneumonia patients, respectively. We trained ViTCNX and the other comparable models using the training set and then evaluated their performance using the test set. Table 2 and Figure 8 show the precision, recall, accuracy, and F1-score values of ViTCNX and the other six models for the three-classification situation.

**Table 2:** Performance of ViTCNX and the other six models under three classification.

**Metrics**	**Precision**	**Recall**	**Accuracy**	**F1-Score**
EfficientNetV2	0.7783	0.4188	0.4526	0.3221
ConvNeXt	0.9562	0.9397	0.9574	0.9473
DenseNet	0.9487	0.9402	0.9560	0.9442
Swin Transformer	0.9259	0.8754	0.9127	0.8957
ResNet-50	0.9369	0.8936	0.9317	0.9100
Vision Transformer	0.9657	**0.9599**	0.9689	0.9627
ViTCNX	**0.9668**	0.9597	**0.9696**	**0.9631**

Bold values means the highest score under this metric.

From Table 2 and Figure 8, we can observe that ViTCNX computed the best precision, accuracy, and F1-score, greatly outperforming the other six models. Although it calculated a relatively lower recall of 0.9597 than Vision Transformer with a recall of 0.9599, the difference is very minor. Particularly, compared with Vision Transformer, ConvNeXt, DenseNet, ResNet-50, Swin Transformer, and EfficientNetV2, ViTCNX computed a F1-score of 0.9631, better by 0.04, 1.58, 1.89, 5.32, 6.74, and 64.11% than the six models, respectively. These results demonstrate that ViTCNX can more accurately classify CT images from COVID-19, from other pneumonia cases, and healthy individuals.

### The confusion matrix analysis

**Figure 9:**
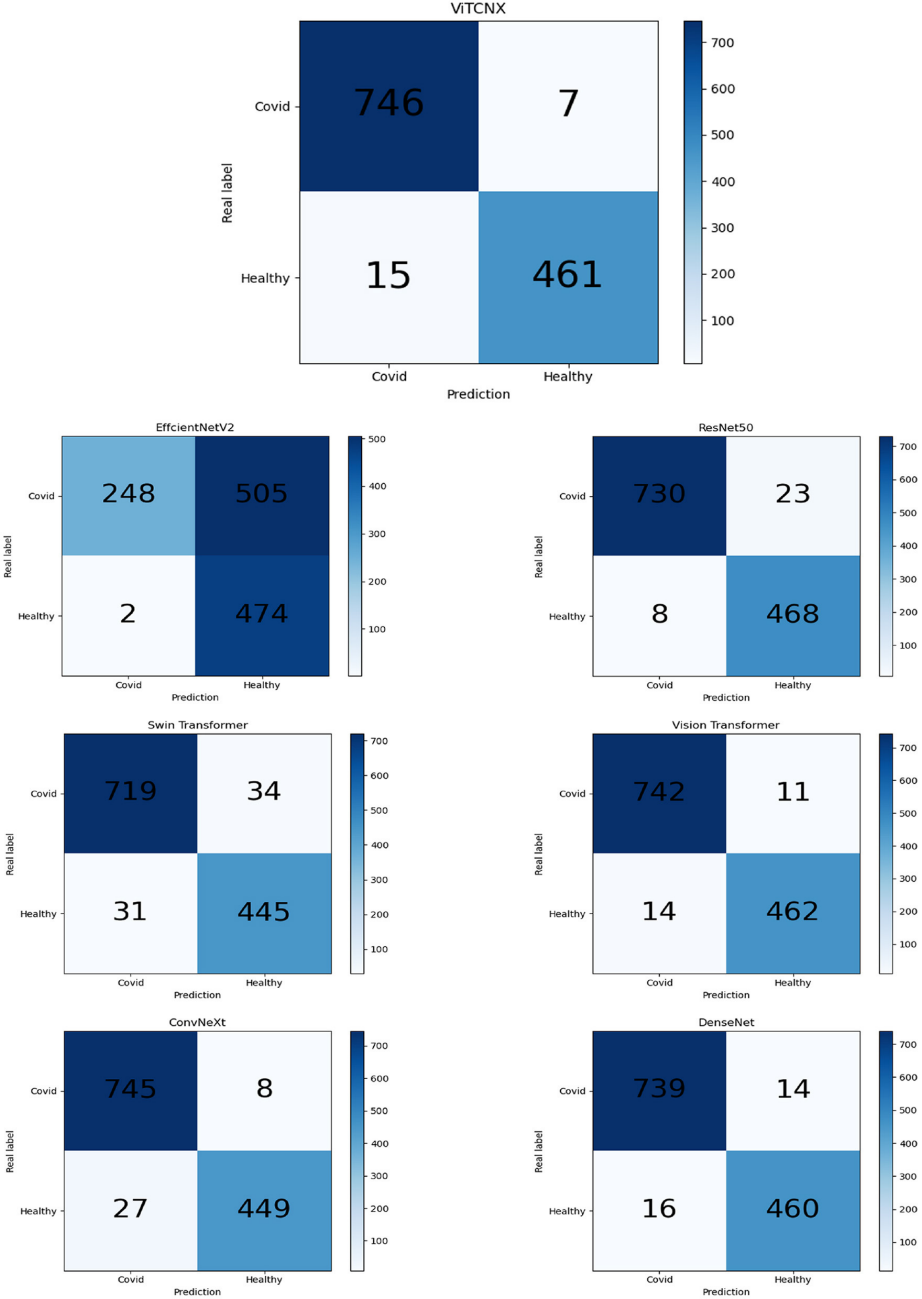
The confusion matrix of results of VitCNX and six other models.

We further evaluated the number of true positives (TP), true negatives (TN), false positives (FP), and false negatives (FN) obtained by Vision Transformer, ConvNeXt, DenseNet, ResNet-50, Swin Transformer, EfficientNetV2, and ViTCNX under binary classification. Table 3 and Figure 9 present the statistical data of TP, TN, FP, and FN from the above seven models for binary classification. The importance of these four evaluation metrics is not equal. For COVID-19 image recognition, TP denotes the number of images that are COVID-19 images in the dataset and are predicted to be COVID-19-related. FN denotes the number of images that are COVID-19 images but are predicted to be non-COVID-19-related. FN denotes that there are undetected COVID-19 patients, which may cause the spread of the pandemic. TP and FN are more important than the other two metrics. Higher TP and lower FN represent the better performance of ViTCNX.

**Table 3:** Statistics of ViTCNX and other six models for binary classification.

**Metrics**	**TP**	**TN**	**FP**	**FN**
EfficientNetV2	248	**474**	**2**	505
ConvNeXt	745	449	27	8
DenseNet	739	460	16	14
Swin Transformer	719	445	31	34
ResNet-50	730	468	8	23
Vision Transformer	742	462	14	11
ViTCNX	**746**	461	15	**7**

Bold values means the highest score under this metric.

From Table 3 and Figure 9, we can observe that our proposed ViTCNX model screens the most TP, and the least FN compared to the other six models. Our proposed ViTCNX model computes the highest TP of 746 and the lowest FN of 7 among 1,229 test samples, demonstrating that it can most efficiently recognize COVID-19-related images of COVID-19 patients.

## Discussion and conclusion

With the rapid development of AI technology and high-performance computing platforms, using deep learning models to detect COVID-19 through lung CT images has become a research hotspot. Not only because this method has a higher performance and faster speed, but also lower time and economic cost. In this paper, we proposed an ensemble deep learning model (ViTCNX) to recognize COVID-19-related CT images by combining Vision Transformer and ConvNeXt. We compared ViTCNX with six other state-of-the-art deep learning models (Vision Transformer, ConvNeXt, DenseNet, ResNet-50, Swin Transformer, and EfficientNetV2). We conducted a series of comparative experiments to evaluate the performance of ViTCNX. The results show that ViTCNX computed the best recall, accuracy, F1-score, AUC, and AUPR under binary classification and the best precision, accuracy, and F1-score under three-classification tests. Moreover, ViTCNX obtained the highest TP and the lowest FN in binary classification. The results show that our proposed ViTCNX model has powerful COVID-19-related image recognition ability.

We adopted several techniques to reduce over-fitting. First, we used three different datasets of COVID-19 to evaluate the performance of ViTCNX. The three datasets were collected from two different places (Wuhan, China, and São Paulo, Brazil). We integrated the three different datasets into one dataset to increase the differences in datasets and further enhance the generalization performance of ViTCNX. Additionally, we used techniques including layer normalization and dropout to prevent over-fitting. The ensemble learning strategies also helped to improve the model's generalization ability and reduce over-fitting.

There are two advantages of the proposed ViTCNX model: First, the variance is reduced through the ensemble of multiple models, thereby improving the robustness and generalization ability of the model. Second, Vision Transformer and ConvNeXt are greatly different in structure. An ensemble of them can lower their correlation and further reduce the classification error. Although ViTCNX obtains better performance, it does increase a large number of training parameters, which increases the training and testing time of the model and requires higher computational resources.

In the future, we will continuously update data to build larger COVID-19 datasets to enhance the generalization ability of ViTCNX. We will also design a new deep learning framework, adopt efficient training methods, and optimize parameter settings to improve the prediction ability of the model. Additionally, we will establish an automatic annotation model to autonomously label hot spots. We anticipate that our proposed ViTCNX model can contribute to the clinical detection of COVID-19.

## Data availability statement

The original contributions presented in the study are included in the article/supplementary material, further inquiries can be directed to the corresponding author/s.

## Author contributions

GT, LP, ZW, XL, and ZL: conceptualization. GT, LP, ZW, CW, and ZL: methodology. ZW, GL, CW, ZH, YZ, and JC: software. GT, LP, ZW, YL, HX, ZH, YZ, and GL: validation. GT, LP, XL, JC, and ZL: investigation. ZW, CW, GL, and HX: data curation. LP and ZW: writing-original draft preparation and project administration. GT and LP: writing-review and editing. GT, LP, and ZL: supervision and funding acquisition. All authors have read and agreed to the published version of the manuscript.

## Funding

ZL was supported by the National Natural Science Foundation of China under Grant No. 62172158. LP was supported by the National Natural Science Foundation of China under Grant No. 61803151. GL and YL were supported by the Innovation and Entrepreneurship Training Program for College Students of Hunan Province under Grant No. S202111535031 and the Innovation and Entrepreneurship Training Program for College Students of the Hunan University of Technology under Grant No. 20408610119.

## Conflict of interest

Author GT was employed by the company Geneis (Beijing) Co., Ltd. Author JC was employed by Hunan Storm Information Technology Co., Ltd. The remaining authors declare that the research was conducted in the absence of any commercial or financial relationships that could be constructed as a potential conflict of interest.

## Publisher's note

All claims expressed in this article are solely those of the authors and do not necessarily represent those of their affiliated organizations, or those of the publisher, the editors and the reviewers. Any product that may be evaluated in this article, or claim that may be made by its manufacturer, is not guaranteed or endorsed by the publisher.
